# Correlation between myeloproliferative diseases and T cell acute lymphoblastic leukemia: a case report and literature review

**DOI:** 10.1016/j.htct.2026.108554

**Published:** 2026-10-15

**Authors:** Viktoria Weihermann, Felipe Vieira Rodrigues Maciel, Elvira DRP Velloso, Luciana Nardinelli, Israel Bendit, Vanderson Rocha, Eduardo Rego, Wellington Fernandes da Silva

**Affiliations:** Department of Hematology, Hospital das Clínicas, Faculdade de Medicina da Universidade de São Paulo, Brazil

## Introduction

Secondary acute myeloid leukemia (AML) evolving from a myeloproliferative neoplasm (MPN) is a well-documented event in the natural history of MPNs. The World Health Organization (WHO) defines this transformation by the presence of ≥20% myeloblasts in the peripheral blood or bone marrow of a patient with a prior history of a myeloproliferative disorder. However, secondary transformation to acute lymphoblastic leukemia (ALL) is exceedingly rare, with only sporadic cases reported in the literature [[1], [2], [3]].

While it is recognized that T lymphocytes and myeloid cells originate from a common hematopoietic progenitor in the bone marrow, the current classification of acute leukemias is based primarily on immunophenotypic characteristics to assign lineage [4,5]. One notable subtype of T-cell acute lymphoblastic leukemia (T-ALL), termed early T-cell precursor (ETP) leukemia, arises from hematopoietic stem cells (HSCs) and exhibits overlapping features with myeloid progenitors [6].

The overlap between T-ALL and myeloid malignancies is acknowledged in rare well-defined entities, such as myeloid/lymphoid neoplasms associated with *PDGFRA, PDGFRB, FGFR1*, or *PCM1::JAK2* rearrangements, as well as in cases of T-lymphoblastic transformation into chronic myeloid leukemia (CML) [4]. Nonetheless, the emergence of T-ALL from Philadelphia chromosome-negative myeloid neoplasms remains exceedingly rare and is poorly characterized in the literature.

Here, we present a brief case report illustrating a peculiar presentation of T-ALL possibly preceded by a myeloproliferative neoplasm, accompanied by unique clinical and genetic features. This report is followed by a comprehensive literature review to contextualize the clinical significance and possible pathogenetic mechanisms underlying this rare phenomenon.

## Case report

A 62-year-old man with a medical history of type 2 diabetes mellitus and hypertension was referred for evaluation due to progressive weight loss and symptoms suggestive of biliary tract obstruction. Symptom onset occurred approximately eight months prior to presentation, with gradual clinical deterioration. Abdominal computed tomography (CT) revealed multiple enlarged lymph nodes, the largest measuring 4 cm in diameter, prompting referral to an oncology center for further investigation. Subsequent positron emission tomography-computed tomography confirmed lymphadenopathy involving cervical, thoracic, and abdominal regions, with a maximum standardized uptake value (SUVmax) of 4.1 in the left retrocrural region. Lymph node biopsy demonstrated infiltration by T-lymphoblastic lymphoma. At that time, a peripheral blood smear examination was unremarkable, with no cytopenias or circulating immature cells.

Bone marrow evaluation revealed 40% blasts with the following immunophenotype: partial cytoplasmic CD3 (46%), CD1a (21%), surface CD3+, CD5+, CD7+++, CD13+, partial CD33, CD34+, CD45++, CD99+++, and partial terminal deoxynucleotidyl transferase (TdT) expression. The blasts were negative for CD2, CD4, CD8, CD19, CD117, myeloperoxidase (MPO), and cytoplasmic IgM (Figure 1). Based on these findings, a diagnosis of early T-cell precursor acute lymphoblastic leukemia (ETP-ALL) was established.

**Figure 1:**
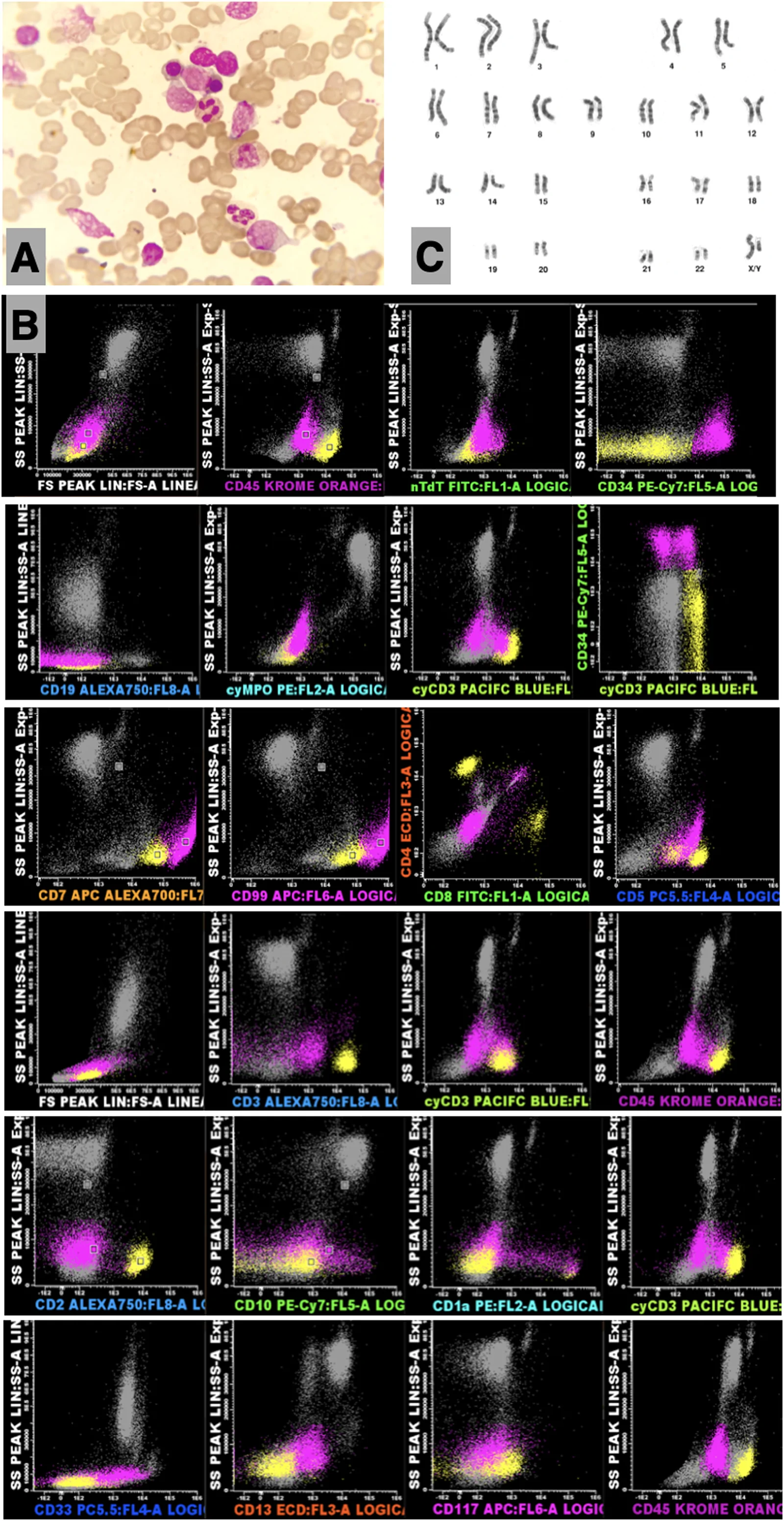
Bone marrow evaluation. A) Aspirate showing blast morphology (Leishman stain, 400X). B) Flow cytometry immunophenotyping: blast population is represented in pink and normal T cells in yellow. C) Metaphase representative of normal karyotype (GTG banding).

Conventional cytogenetic analysis of the bone marrow demonstrated a normal karyotype (Figure 1). A targeted next-generation sequencing panel encompassing 40 genes and 27 fusion genes (Ion Torrent, Oncomine Myeloid Assay Panel) commonly implicated in leukemias identified three oncogenic variants, as detailed in Table 1. Based on these findings, a comprehensive review of the patient’s medical history was conducted, revealing two notable features: an episode of unprovoked deep vein thrombosis approximately ten years earlier and a documented platelet count of 883 × 10^3^/µL one year prior to diagnosis. Collectively, these observations raised the suspicion of a previously undiagnosed essential thrombocythemia. Unfortunately, the patient had not been followed at our institution prior to referral, which occurred due to a suspicion of leukemia. Consequently, sufficiently detailed information regarding prior clinical events is unavailable, precluding confirmation of a pre-existing myeloproliferative disorder.

**Table 1:** Oncogenic variants.

Gene	Locus	Amino Acid Change	VAF (%)	Type of variant	Variant ID
*NRAS*	chr1:115258747	p.Gly12Asp	8.78	Missense	COSM564
*JAK2*	chr9:5073770	p.Val617Phe	5.0	Missense	COSM12600
*IDH2*	chr15:90631934	p.Arg140Gln	25	Missense	COSM41590

VAF: Variant Allele Frequency

The patient subsequently initiated treatment with an adapted acute lymphoblastic leukemia regimen for older adults (Elderly GRAALL), as previously reported by our group [7]. The initial cerebrospinal fluid (CSF) evaluation showed no evidence of central nervous system involvement. Following the two-block induction phase, the patient achieved complete cytomorphological remission; however, measurable residual disease (MRD) remained positive at 1.73%. Consolidation therapy was administered as planned, but MRD levels increased to 2.89% after the first consolidation block.

Second-line treatment with FLAG-daunorubicin (fludarabine, cytarabine, granulocyte colony-stimulating factor and daunorubicin) was initiated in January 2024. Post-induction assessment again demonstrated persistent MRD positivity (0.8%). Consequently, third-line therapy with venetoclax in combination with low-dose cytarabine (LDAC) was initiated. After two treatment cycles, a reevaluation demonstrated MRD negativity.

The patient subsequently underwent haploidentical related-donor hematopoietic stem cell transplantation using a reduced-intensity conditioning regimen consisting of cyclophosphamide, fludarabine, and total body irradiation. Graft-versus-host disease (GVHD) prophylaxis included post-transplant cyclophosphamide, cyclosporine, and mycophenolate mofetil. Bone marrow assessment on Day +30 demonstrated MRD negativity with complete (100%) donor chimerism. However, by Day +100, donor chimerism had decreased to 56%, accompanied by bone marrow relapse with 37% blasts. Given the very early post-transplant relapse, salvage therapy with low-dose cytarabine plus venetoclax was attempted. Unfortunately, the patient developed septic shock and died a few days later.

## Discussion

Early T-cell precursor acute lymphoblastic leukemia is a highly aggressive subtype of T-ALL. It is characterized by the aberrant expression of myeloid/stem cell antigens, and the lack of some T-lymphoid antigens, including CD1a, CD4, CD5, and CD8. Some authors consider these immature leukemias (both myeloid and lymphoid) as a spectrum, since these entities have close, or sometimes overlapping characteristics [8].

In addition, the gene expression signature of ETP-ALL demonstrates a higher frequency of mutations commonly associated with myeloid neoplasms, such as *FLT3, DNMT3A*, and *IDH1* [9]. Notably, *JAK* mutations are detected in approximately 20–30% of T-ALL cases, predominantly in ETP-ALL, leading to constitutive activation of the JAK/STAT signaling pathway [10]. However, *JAK2 V617F* has not been described as one of the frequent mutations in T-ALL. In a Japanese cohort, this mutation was not detected in lymphoid malignancies [11]. Despite that, this variant is a well-established driver mutation in myeloproliferative neoplasms and it is also observed in clonal hematopoiesis of indeterminate potential (CHIP).

The *NRAS* variant identified in our patient corresponds to a single-nucleotide polymorphism previously described in childhood ALL [12] and is classified as oncogenic according to genetic databases such as Franklin. Finally, the *IDH2* variant identified in our patient has been frequently reported in acute myeloid leukemia. In a study evaluating the clinical impact of *IDH1/IDH2* mutations in T-ALL, *IDH2* mutations were observed in 3% of cases, with R140Q representing the most prevalent variant (78%) [13].

From a therapeutic perspective, although B- and T-cell leukemias are molecularly and clinically distinct, treatment approaches have largely remained similar to date. Nevertheless, several small studies have explored the incorporation of novel agents into T-ALL treatment, including drugs traditionally used in acute myeloid leukemia.

Given that BCL-2 overexpression has been widely reported in ALL, inhibitors of the BCL-2 family represent a promising therapeutic strategy. In a phase II trial, venetoclax combined with azacitidine was administered to eighteen patients with relapsed or refractory T-cell leukemia [14]. The overall response rate was 88.9%, and with a median follow-up of 24.1 months, the estimated median overall survival was 24.1 months [14]. After two treatment cycles, 22% of patients, similar to our case, achieved complete remission. The relapse rate in this cohort was 27.8%. Notably, several additional studies evaluating venetoclax and navitoclax in ALL have reported favorable outcomes [15].

Another potential therapeutic target involves the use of *JAK* inhibitors. In a preclinical study, ruxolitinib combined with dexamethasone for the mutant IL-7 T-ALL cell line DND-41 resulted in a significant, dose-dependent reduction in cellular proliferation compared with dexamethasone alone [16]. Furthermore, a case report of refractory JAK3-mutated T-cell leukemia demonstrated clinical benefit with ruxolitinib therapy. In this case, the patient received ruxolitinib at a dose of 10 mg twice daily following relapse after allogeneic hematopoietic stem cell transplantation, achieving complete remission of skin lesions and full donor chimerism, although minimal residual disease remained detectable (1 × 10⁻⁵) [17].

Analysis of the patient’s genetic profile also identified *IDH* mutations as a potential therapeutic target. A case report described a patient with B-ALL harboring an *IDH1* R132H mutation who was treated with ivosidenib at relapse and remained in second remission for one year [18]. Regarding enasidenib, no reports were identified in ALL; however, an ongoing clinical trial at Memorial Sloan Kettering Cancer Center is currently evaluating its use in angioimmunoblastic T-cell lymphoma [19].

In summary, this case underscores the importance of comprehensive myeloid mutation profiling in T-ALL, as the identification of such alterations may expand therapeutic options and potentially improve clinical outcomes.

## Data availability statement

The data that support the findings of this study are available from the corresponding author upon reasonable request.

## Conflicts of interest

None.
